# Metabolic discrimination of sea buckthorn from different *Hippophaë* species by ^1^H NMR based metabolomics

**DOI:** 10.1038/s41598-017-01722-3

**Published:** 2017-05-08

**Authors:** Yue Liu, Gang Fan, Jing Zhang, Yi Zhang, Jingjian Li, Chao Xiong, Qi Zhang, Xiaodong Li, Xianrong Lai

**Affiliations:** 1 College of Ethnic Medicine, Chengdu University of Traditional Chinese Medicine, Chengdu, 611137 China; 2 National Institute for Food and Drug Control, Beijing, 100050 China; 3 Sichuan Industrial Institute of Antibiotics, Chengdu University, Chengdu, 610051 China; 4 College of Forestry and Landscape Architecture, South China Agricultural University, Guangzhou, 510642 China; 5 College of Pharmacy, Hubei University of Chinese Medicine, Wuhan, 430065 China

## Abstract

Sea buckthorn (*Hippophaë*; Elaeagnaceae) berries are widely consumed in traditional folk medicines, nutraceuticals, and as a source of food. The growing demand of sea buckthorn berries and morphological similarity of *Hippophaë* species leads to confusions, which might cause misidentification of plants used in natural products. Detailed information and comparison of the complete set of metabolites of different *Hippophaë* species are critical for their objective identification and quality control. Herein, the variation among seven species and seven subspecies of *Hippophaë* was studied using proton nuclear magnetic resonance (^1^H NMR) metabolomics combined with multivariate data analysis, and the important metabolites were quantified by quantitative ^1^H NMR (qNMR) method. The results showed that different *Hippophaë* species can be clearly discriminated and the important interspecific discriminators, including organic acids, L-quebrachitol, and carbohydrates were identified. Statistical differences were found among most of the *Hippophaë* species and subspecies at the content levels of the aforementioned interspecific discriminators via qNMR and one-way analysis of variance (ANOVA) test. These findings demonstrated that ^1^H NMR-based metabolomics is an applicable and effective approach for simultaneous metabolic profiling, species differentiation and quality assessment.

## Introduction

Sea buckthorn (*Hippophaë*), from the Elaeagnaceae family, is a wild or cultivated shrub and mainly distributed in cold arid regions throughout Europe and Asia^1, 2^. Previous reports showed that seven species and 11 subspecies of the genus *Hippophaë* have been recognized worldwide based on morphological variations^3–7^. As demonstrated in recent scientific studies and clinical trials, the ripe berries of sea buckthorn are the potential source of bioactive substances, including vitamins, carotenoids, phytosterols, organic acids, fatty acids, free amino acids, and a variety of flavonoids^8, 9^. On account of these powerful bioactive phytochemicals, which have a wide spectrum of medicinal and nutritional effects, such as antioxidant, immunomodulatory, anti-atherogenic, anti-stress, hepatoprotective, radioprotective, and tissue repair activities^1, 9–13^, sea buckthorn berries are used in food, fresh juice, beverages, nutraceutical products, and cosmetics. In addition to being used as food source, sea buckthorn berries have been used in Chinese ethnic medicine since Tang Dynasty (618–907 AD), to treat various human diseases, such as skin injuries, mucous membranes injuries of stomach and, lung disorders, cardiovascular disease and high altitude diseases^2, 14, 15^.

As a commonly used nutritional supplement and ethnic medicine, sea buckthorn berries have been recorded in the state standard, local standards, and the List of Medicinal and Edible Plant of China, as well as the Ayurvedic Pharmacopoeia of India^15–19^. However, the plant origins differed in these standards for the treatment of different diseases, even for the same disease. The count shows that three species and five subspecies (*H*. *rhamnoides*, *H*. *gyantsensis* and *H*. *tibetana*) can be used as medicinal materials. Thus, confusion has arisen because the common name is used for a number of *Hippophaë* taxa that have similar morphology. It has been reported that different *Hippophaë* species vary in their secondary metabolites spectra, which results in a variation in treatment and health care efficacies^2, 14, 20, 21^. In addition, different species and subspecies have their own specific outstanding properties, such as fruit quality, ease to harvest and process, and drought and cold resistance abilities, especially related to the treatment of various diseases^21^.

Plant metabolomics has recently attracted considerable attention because the method focuses on the holistic characteristics, and has been developed as an important approach for the modern research of (medicinal) plants. LC/GC-MS and ^1^H NMR are the most commonly used analytical strategies in metabolomics^22–26^. Compared with the two chromatographic techniques, ^1^H NMR based metabolomics is considered as the most promising analytical tool because the method allows simultaneously detection of primary and secondary metabolites in a single run, and produces a non-biased abundant metabolic profile. Additionally, this method also can be used to analyze various classes of chemical constituents both qualitatively and quantitatively, with simple sample preparation steps and good reproducibility^27^. Thus, ^1^H NMR based metabolomics combined with multivariate data analysis has recently demonstrated its suitability for metabolomics studies for the discrimination, authentication, and quality assessment of food, crops and herbal medicines^28–32^.

In a recent study, three origins of sea buckthorn berries (*H*. *rhamnoides* ssp. *sinensis*, *H*. *gyantsensis*, and *H*. *tibetana*) were investigated through ^1^H NMR based metabolomics. The results indicated that three species can be separated and some species discriminators were identified^14, 20^. However, the developed method has not been applied to large sample populations, and the differences in metabolites of sea buckthorn berries among *Hippophaë* species are unclear. Besides, the number of identified metabolites from ^1^H NMR spectra was inadequate, and some of the metabolites which have great significance to the quality evaluation, were not quantified. Therefore, the present study aims to discriminate sea buckthorn berry samples from all seven species and seven subspecies of *Hippophaë* native to China, identify and quantify the potential discriminating metabolites by using ^1^H NMR based metabolomics. Additionally, ^1^H NMR spectroscopy coupled with multivariate statistical analysis will serve as a useful method for comparison and quality evaluation of sea buckthorn berries from different *Hippophaë* species both accurately and reliably.

## Results

### ^1^H NMR spectra inspection and metabolites identification

**Figure 1:**
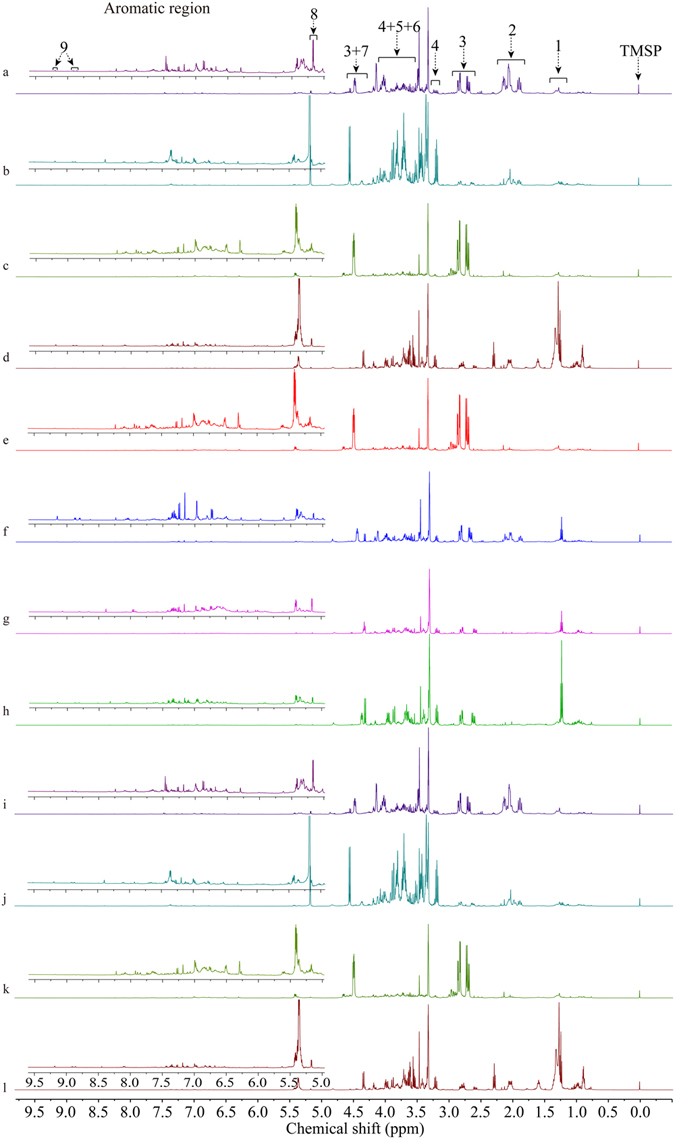
Representative ^1^H NMR spectra of berry sample extracts from seven species and seven subspecies of *Hippophaë* (0.0~9.5 ppm). (1) fatty acids, (2) quinic acid, (3) malic acid, (4) glucose, (5) L-quebrachitol, (6) D-fructose, (7) *β*-D-glucose, (8) *α*-D-glucose, (9) trigonelline, (**a**) *H*. *rhamnoides* ssp. *sinensis*, (**b**) *H*. *rhamnoides* ssp. *mongolica*, (**c**) *H*. *rhamnoides* ssp. *yunnanensis*, (**d**) *H*. *rhamnoides* ssp. *turkestanica*, (**e**) *H*. *rhamnoides* ssp. *wolongensis*, (**f**) *H*. *goniocarpa*, (**g**) *H*. *litangensis*, (**h**) *H*. *neurocarpa* ssp. *neurocarpa*, (**i**) *H*. *neurocarpa* ssp. *stellatopilosa*, (**j**) *H*. *salicifolia*, (**k**) *H*. *gyantsensis*, and (**l**) *H*. *tibetana*.

A general understanding of the representative ^1^H NMR spectra of the berry sample methanol-water extracts from different *Hippophaë* species and subspecies was provided in Fig. 1. As seen from the spectra, the metabolic profile of sea buckthorn berries from different botanical origins showed similar outlines, fatty acids, organic acids, L-quebrachitol, and glucose were their common metabolites. However, the metabolite intensities varied a lot, such as the signal intensity of malic acid in *H*. *rhamnoides* ssp. *yunnanensis* and *H*. *rhamnoides* ssp. *wolongensis* were obvious higher than that in other species, and *H*. *rhamnoides* ssp. *mongolica* showed the highest level of reducing sugar (*β*-D-glucose and *α*-D-glucose) among *Hippophaë* species from different botanical origins.

Although the aromatic region in ^1^H NMR spectra of all investigated species showed lower signal intensities, the differences of signal intensities still have been revealed by expanding the spectra (Fig. 1), for example, *H*. *rhamnoides* ssp. *wolongensis* and *H*. *goniocarpa* displayed higher intensities of phenolic compounds. Thus, some of the *Hippophaë* species can be visually identified. By comparing the ^1^H NMR spectra of the standard compounds, adding relevant reference compounds directly to a pH-adjusted NMR sample, and comparisons to the published literature^8, 14, 20, 33–36^, a total of 36 metabolites were finally identified. The detailed information for the assigned peaks can be found in Table 1 and Supplementary Fig. S1.

**Table 1:** Identification of metabolites in berry samples from seven species and seven subspecies of *Hippophaë* by ^1^H NMR spectroscopy.

Metabolites	Multiplicity^a^, chemical shifts (ppm), *J* (Hz)
Saturated fatty acids	0.87 (m), 1.28 (m), 1.58 (m), 2.52 (dd, *J*_*1*_ = 9.2, *J*_*2*_ = 5.6 Hz)
Unsaturated fatty acids	0.87 (m), 1.28 (m), 1.58 (m), 2.28 (m), 2.52 (dd, *J*_*1*_ = 9.2, *J*_*2*_ = 5.6 Hz), 5.33 (m)
Leucine	0.97 (d, *J* = 5.1 Hz)
Valine	1.00 (d, *J* = 4.9 Hz), 1.02 (d, *J* = 7.1 Hz)
Alanie	1.49 (d, *J* = 7.2 Hz)
Quinic acid	1.87 (dd, *J*_*1*_ = 13.0, *J*_*2*_ = 11.0 Hz), 2.04 (m), 4.16 (m)
Malic acid	2.68 (dd, *J*_*1*_ = 16.2, *J*_*2*_ = 7.4 Hz), 2.82 (dd, *J*_*1*_ = 16.2, *J*_*2*_ = 4.5 Hz), 4.46 (dd, *J*_*1*_ = 7.2, *J*_*2*_ = 4.6 Hz)
L-quebrachitol	3.45 (s), 3.63 (m)
D-fructose	3.57 (m), 3.70 (m), 3.78 (m), 4.01 (m), 4.10 (m)
Isoleucine	1.00 (d, *J* = 4.9 Hz), 1.02 (d, *J* = 7.1 Hz)
Dehydroascorbic acid	4.60 (m)
Sterols	0.68 (s)
Oleanolic acid	0.776 (d, *J* = 9.9 Hz), 0.93 (m), 1.11 (s), 5.25 (s)
Sucrose	5.39 (d, *J* = 3.8 Hz)
Uridine	5.90 (d, *J* = 8.1 Hz), 5.91 (s), 7.90 (d, *J* = 2.0 Hz)
Tryptophan	7.76 (d, *J* = 2.2 Hz)
Histidine	8.66 (m)
Trigonelline	8.84 (brd, *J* = 5.8 Hz), 8.89 (brd, *J* = 8.4 Hz), 9.17 (brs)
*β*-D-glucose	3.20 (m), 3.89 (m), 4.53 (d, *J* = 7.8 Hz)
*α*-D-glucose	3.20 (m), 3.52 (dd, *J*_*1*_ = 4.5, *J*_*2*_ = 10.1), 5.15 (d, *J* = 3.7 Hz)
Asparagine	2.91 (d, *J* = 3.8 Hz), 2.94 (d, *J* = 3.9 Hz)
Quercetin	6.25 (s), 6.44 (d, *J* = 5.3 Hz), 6.94 (m), 7.59 (s), 7.63 (d, *J* = 2.0 Hz), 7.73 (d, *J* = 2.1 Hz)
Kaempferol	6.25 (s), 6.44 (d, *J* = 5.3 Hz), 6.94 (m), 7.73 (s), 8.06 (d, *J* = 2.4 Hz)
Isorhamnetin	3.81 (s), 6.25 (s), 6.44 (d, *J* = 5.3 Hz), 6.93 (d, *J* = 4.1 Hz), 6.97 (s), 7.67 (d, *J* = 2.2 Hz)
Quercetin-3-*O*-*β*-D-rutinoside	6.28 (d, *J* = 2.0 Hz), 6.48 (d, *J* = 1.8 Hz), 6.93 (d, *J* = 4.1 Hz), 7.59 (s), 7.67 (d, *J* = 2.2 Hz)
Quercetin-3-*O*-*β*-D-glucoside	6.28 (d, *J* = 2.0 Hz), 6.48 (d, *J* = 1.8 Hz), 6.93 (d, *J* = 4.1 Hz), 7.59 (s), 7.73 (d, *J* = 2.1 Hz)
Isorhamnetin-3-*O*-*β*-D-rutinoside	1.08 (d, *J* = 6.0 Hz), 3.81 (s), 6.28 (d, *J* = 2.0 Hz), 6.48 (d, *J* = 1.8 Hz), 6.93 (d, *J* = 4.1 Hz), 7.90 (d, *J* = 2.0 Hz)
Isorhamnetin-3-*O*-*β*-D-glucoside	3.81 (s), 6.28 (d, *J* = 2.0 Hz), 6.48 (d, *J* = 1.8 Hz), 6.93 (d, *J* = 4.1 Hz), 7.59 (s), 7.90 (d, *J* = 2.0 Hz)
Isorhamnetin-3-*O*-*β*-D-glucoside-7-*O*-*α*-L-rhamnoside	2.99 (m), 4.78 9 (m), 5.61 (brs), 6.48 (d, *J* = 1.8), 6.97 (d, *J* = 1.8), 7.59 (s), 7.95 (m)
Quercetin-3-*O*-*β*-D-glucoside-7-*O*-*α*-L-rhamnoside	5.39 (d, *J* = 3.8 Hz), 6.48 (d, *J* = 1.8 Hz), 6.81 (d, *J* = 5.5 Hz), 6.93 (d, *J* = 4.1 Hz), 7.49 (d, *J* = 7.4), 7.73 (d, *J* = 2.1 Hz), 7.90 (d, *J* = 2.0 Hz), 7.95 (m)
Isorhamnetin-3-*O*-*β*-D-galactoside-7-*O*-*α*-L-rhamnoside	2.99 (m), 6.48 (d, *J* = 1.8 Hz), 6.97 (d, *J* = 1.8 Hz), 7.59 (s), 7.95 (m)
Isorhamnetin-3-*O*-*α*-L-arabinopyranoside-7-*O*-*α*-L-rhamnoside	3.17 (s), 5.61 (brs), 6.48 (d, *J* = 1.8 Hz), 6.93 (d, *J* = 4.1), 7.64 (dd, *J*_*1*_ = 8.5 Hz, *J*_*2*_ = 2.1 Hz)
Kaempferol-3*-O*-*β*-D-sophoroside-7-*O*-*α*-L-rhamnoside	6.49 (d, *J* = 2.1 Hz), 8.06 (d, *J* = 2.4 Hz)
Isorhamnetin-3-*O*-*β*-D-sophoroside-7-*O*-*α*-L-rhamnoside	6.48 (d, *J* = 2.1 Hz), 6.81 (d, *J* = 5.5 Hz), 6.97 (d, *J* = 1.8 Hz)
Quercetin-7-*O*-*α*-L-rhamnoside	5.61 (brs), 6.48 (d, *J* = 1.8 Hz), 7.64 (dd, *J*_*1*_ = 8.5 Hz, *J*_*2*_ = 2.1 Hz)
Isorhamnetin-7-*O*-*α*-L-rhamnoside	5.61 (brs), 6.48 (d, *J* = 1.8 Hz)

^a^Multiplicity: s, singlet; d, doublet; t, triplet; and m, multiplet.

### Multivariate statistical analysis

**Figure 2:**
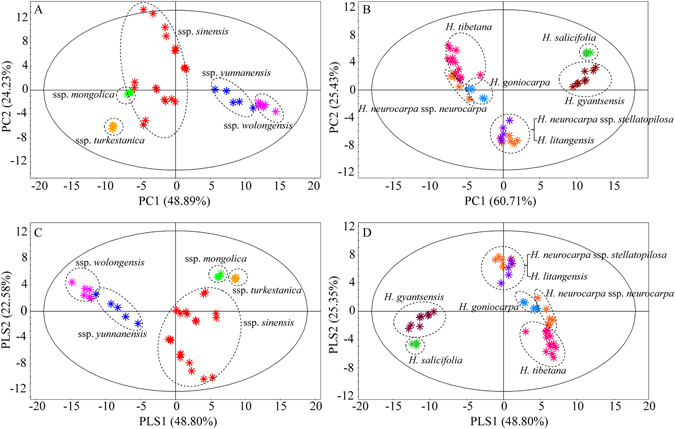
Multivariate model plots of the ^1^H NMR data. (**A**) PCA score plot of five *H*. *rhamnoides* subspecies, (**B**) PCA score plot of the remaining six *Hippophaë* species, (**C**) PLS-DA score plot of five *H*. *rhamnoides* subspecies, (**D**) PLS-DA score plot of the remaining six *Hippophaë* species.

Principle component analysis (PCA) on the ^1^H NMR spectral data was used to compare interpretations of different variations of sea buckthorn berries, visualize the underlying trend, and understand the metabolic differentiation among sea buckthorn berries from different *Hippophaë* species. PCA was first performed on ^1^H NMR spectral data of all 90 samples (Supplementary Fig. S2). However, noticeable overlaps were found among different species, indicating that these samples could not be well separated. Therefore, the closely related five subspecies belonging to *H*. *rhamnoides*, and the remaining six *Hippophaë* species samples were separately analyzed for an unambiguous discrimination. The PCA scores plot (PC1 = 48.89%, PC2 = 24.23%) of the mean-centred dataset in Fig. 2A showed clear discrimination of samples based on five subspecies of *H*. *rhamnoides*. Samples of *H*. *rhamnoides* ssp. *sinensis* were projected in a larger region compared with the other four *H*. *rhamnoides* subspecies, indicating that the intraspecific variations in *H*. *rhamnoides* ssp. *sinensis* were larger than the variations in the four *H*. *rhamnoides* subspecies. However, samples of five *H*. *rhamnoides* subspecies can be clearly classified. The PCA result of the remaining six *Hippophaë* species was shown in Fig. 2B. Samples belong to each species were significantly differentiated, only except samples of *H*. *litangensis* and *H*. *neurocarpa*.

Partial least squares discriminant analysis (PLS-DA), a supervised approach, sharpen the separation between groups of observations by using the class information^37^. The PLS-DA score plots provided good agreement with the result of the PCA (Fig. 2C and D). The PLS-DA score plot of *H*. *rhamnoides* subspecies showed that five subspecies can be clearly separated (R^2^X_(cum)_ = 0.988, R^2^Y_(cum)_ = 0.934, and Q^2^_(cum)_ = 0.843). The model was validated with 200 permutations of eight components, and had a proper R^2^Y-intercept of 0.16 and Q^2^Y-intercept of −0.66 (Supplementary Fig. S3A). The PLS-DA model of the remaining six *Hippophaë* species was also validated with 200 permutations of 13 components (R^2^Y-intercept = 0.39, Q^2^Y-intercept = −1.28), and the classification results are consistent with the PCA results (R^2^X _(cum)_ = 0.995, R^2^Y_(cum)_ = 0.952, and Q^2^_(cum)_ = 0.747), (Supplementary Fig. S3B).

**Figure 3:**
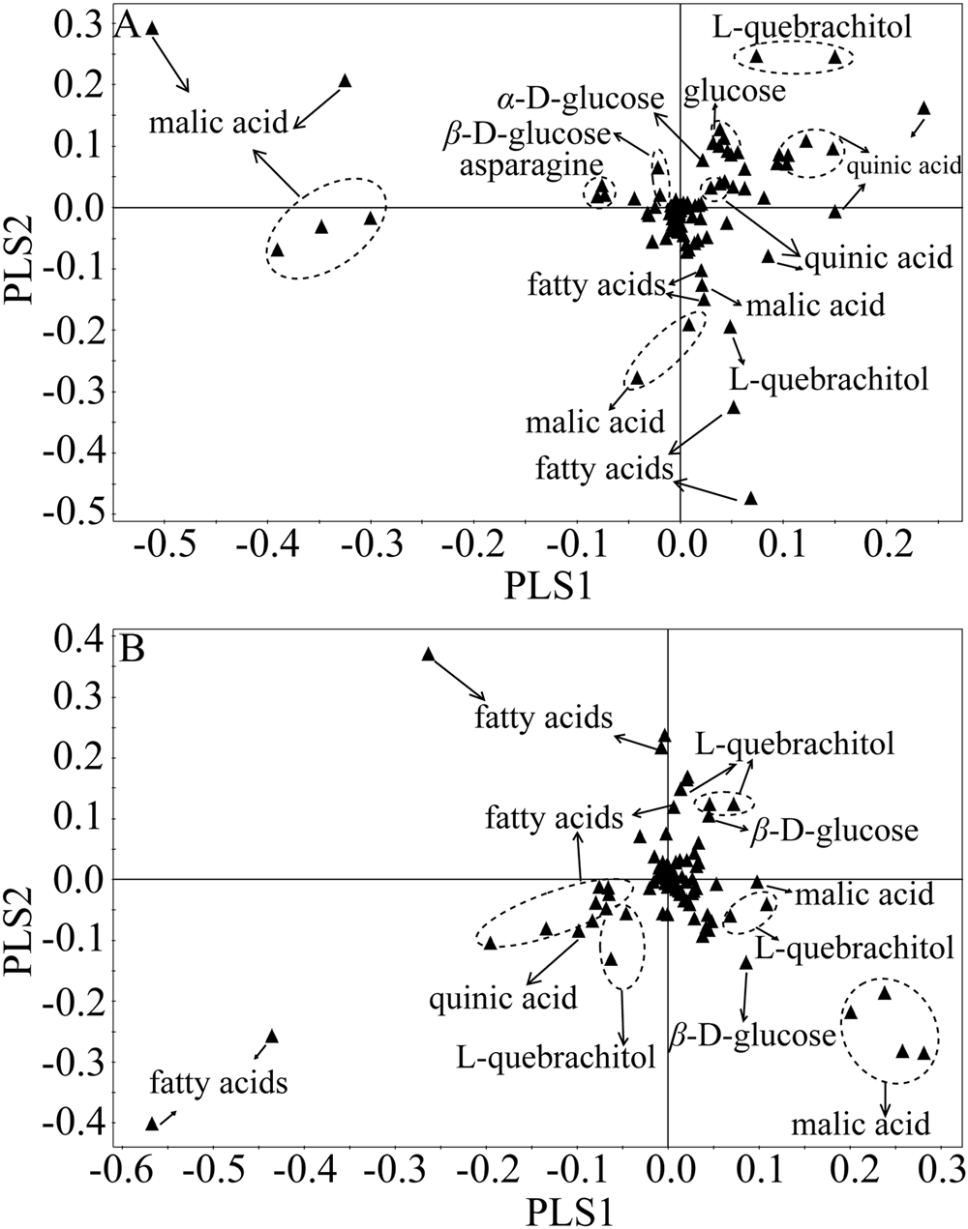
PLS-DA loading plots of (**A**) five *H*. *rhamnoides* subspecies and (**B**) the remaining six *Hippophaë* species.

The corresponding loading plots of PLS-DA elucidated that signals from quinic acid, malic acid, L-quebrachitol, glucose, and fatty acids were the dominant discriminators at both the subspecies and species level (Fig. 3). Moreover, the different levels of metabolites in sea buckthorn berries were found to be associated with its quality in PLS-DA loading plots at both the subspecies and species level. Malic acid level was higher in *H*. *rhamnoides* ssp. *yunnanensis* and *H*. *rhamnoides* ssp. *wolongensis* than that in other three *H*. *rhamnoides* subspecies. L-quebrachitol level in *H*. *rhamnoides* ssp. *mongolica* and *H*. *rhamnoides* ssp. *turkestanica* were higher than that in the other three *H*. *rhamnoides* subspecies. Fatty acids and malic acid levels in *H*. *rhamnoides* ssp. *sinensis* were higher than those in *H*. *rhamnoides* ssp. *mongolica* and *H*. *rhamnoides* ssp. *turkestanica* (Fig. 3A). The PLS-DA loading plot of the remaining of six *Hippophaë* species illustrated that glucose, L-quebrachitol and malic acid were higher in *H*. *tibetana* than those in other species (Fig. 3B).

### Metabolites quantification

**Figure 4:**
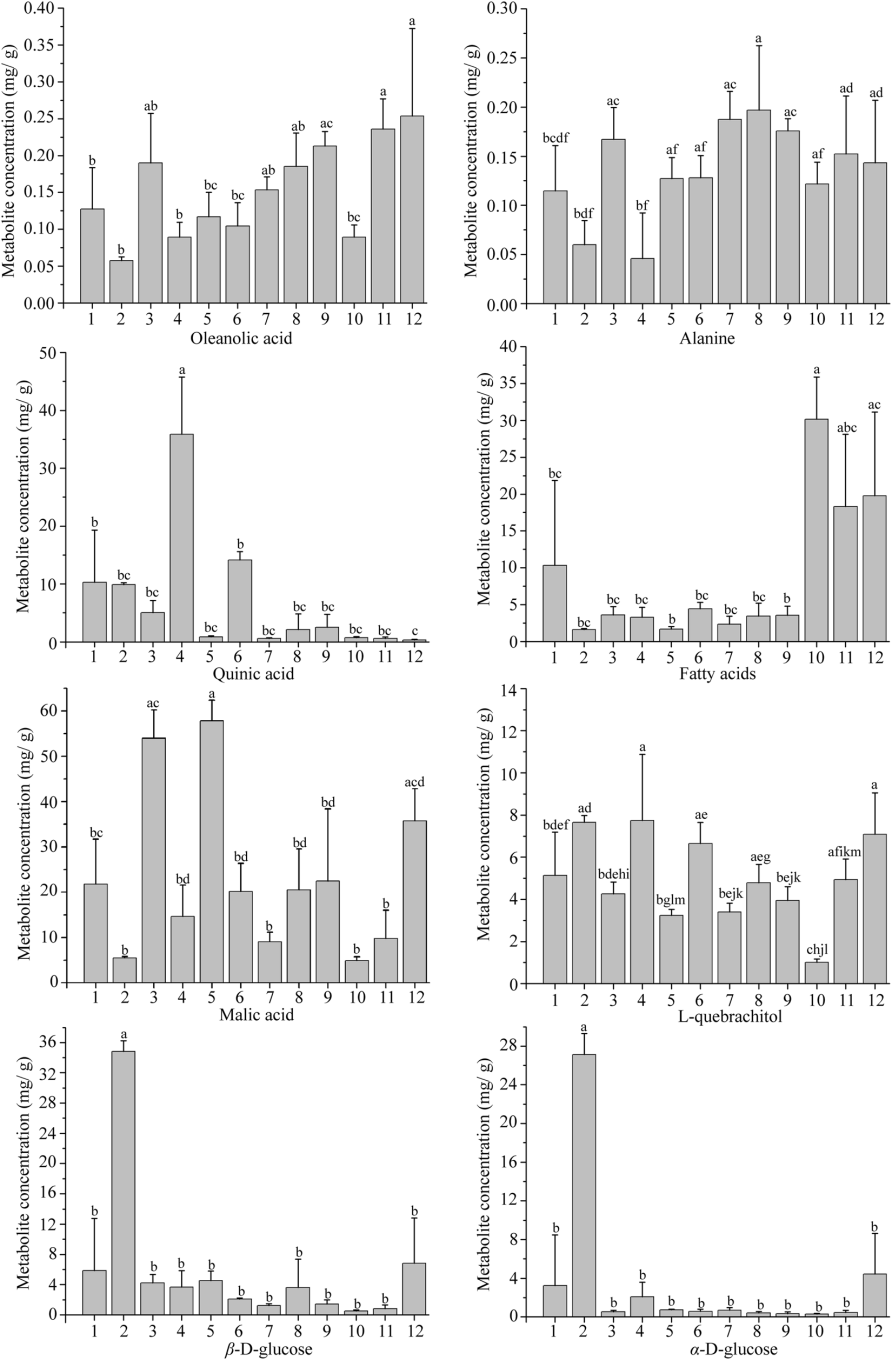
^1^H NMR intensities of eight metabolites in sea buckthorn berries from seven species and seven subspecies of *Hippophaë*. (1) *H*. *rhamnoides* ssp. *sinensis*, (2) *H*. *rhamnoides* ssp. *mongolica*, (3) *H*. *rhamnoides* ssp. *yunnanensis*, (4) *H*. *rhamnoides* ssp. *turkestanica*, (5) *H*. *rhamnoides* ssp. *wolongensis*, (6) *H*. *goniocarpa*, (7) *H*. *litangensis*, (8) *H*. *neurocarpa* ssp. *neurocarpa*, (9) *H*. *neurocarpa* ssp. *stellatopilosa*, (10) *H*. *salicifolia*, (11) *H*. *gyantsensis*, and (12) *H*. *tibetana*. Data are given as mean ± SD. Different letters above the bars indicate significant differences between species, and the same letters above the bars indicate no significant differences between species based on Tukey’s multiple comparison tests (*p* < 0.05).

To obtain a better understanding of the different individual metabolite levels among berry samples from seven species and seven subspecies of *Hippophaë*, eight selected metabolites were quantitatively analyzed through qNMR method. The characteristic signals of oleanolic acid (0.75 ppm, d), alanine (1.49 ppm, d), quinic acid (1.89 ppm, dd), fatty acids (2.27 ppm, m), malic acid (2.68 ppm, dd), L-quebrachitol (3.45 ppm, s), *β*-D-glucose (4.53 ppm, d), *α*-D-glucose (5.15 ppm, d), were selected for quantification, and the results are shown in Fig. 4 and Supplementary Table S1. Statistical differences were observed among most of the *Hippophaë* species and subspecies in the level of all selected metabolites (*p* < 0.05) (Fig. 4). The concentration of fatty acids in *H*. *rhamnoides* ssp. *sinensis*, *H*. *salicifolia*, *H*. *gyantsensis* and *H*. *tibetana* were significantly higher than in other *Hippophaë* species. The level of alanine in *H*. *rhamnoides* ssp. *mongolica* and *H*. *rhamnoides* ssp. *turkestanica* were lower than that in other species. The level of malic acid in *H*. *rhamnoides* ssp. *yunnanensis* and *H*. *rhamnoides* ssp. *wolongensis* were obviously higher than those in other species. Quinic acid level in *H*. *rhamnoides* ssp. *turkestanica* was higher than in that the other species. *H*. *salicifolia* contained the lowest level of L-quebrachitol, and *H*. *rhamnoides* ssp. *mongolica* contained the highest level of *α*-D-glucose and *β*-D-glucose among all *Hippophaë* species. These findings were in good agreement with the PLS-DA results.

## Discussion

As an important ecologically and economically plant, sea buckthorn has attracted international attention and serves as a store house for researchers in the field of biotechnology, nutraceutical, pharmaceutical, cosmetic, environmental and other disciplines^10^. It is said that the ripe berries must be harvested at the correct stage and frozen immediately or processed quickly for its best taste and bioactive components retention^38^. Then it can be dried, pressed or extracted for the uses of raw medicinal and nutritional materials, juice and other purposes^9, 11^. Thus, sea buckthorn berries are facing an increasing demand along with the improvement of species identification, and quality control method. The adequate medicinal and nutritional value of the correct species and high-quality of raw materials are important to ensure the safety and efficacy of commercial food and medicine products.

In this study, the chemical profiling and species discrimination of sea buckthorn berries from seven species and seven subspecies of *Hippophaë* native to China were achieved using ^1^H NMR based metabolomics coupled with multivariate statistical analysis, and quantitation of major metabolites that were used to discriminate species was carried out using TMSP as reference. Moreover, differences of metabolite signal intensities were observed among *Hippophaë* species, such as quinic acid, malic acid, L-quebrachitol, *β*-D-glucose, and *α*-D-glucose. Further PLS-DA loading plots and qNMR method coupled with ANOVA proved that these metabolites were important species and subspecies discriminators.

Although fatty acids were revealed in the PCA and PLS-DA loading plots as species and subspecies discriminator, the extraction solvent this study used was favor of polar compounds. Therefore, the quantification of fatty acids may not reflect the real content in the raw material, and this metabolite cannot be regarded as a metabolic marker for species/subspecies differentiation in the present study. Moreover, due to the low signal intensity of aromatic region in ^1^H NMR spectra, some useful information was covered by the metabolites with strong signal intensity. Thus, this region (6.5~9.5 ppm) was separately analyzed to investigate the differences among *Hippophaë* species. The data set was also imported into SIMCA-P and a Pareto scaling method was applied to reduce the influence of the intense peaks in this region. The PCA and PLS-DA score plots showed that most of the investigated *Hippophaë* species/subspecies can be clearly classified, and the loading plots illustrated that the discriminator in aromatic region were phenolic compounds, such as quercetin, kaempferol, isorhamnetin, and quercetin-3-*O-β*-D-rutinoside (Supplementary Fig. S4). Hence, it can be concluded that primary metabolites, such as organic acids, sugars, L-quebrachitol, and secondary metabolites, like flavonoid aglycones and flavonoid glycosides were both metabolic markers for species/subspecies discrimination.

Sugars, organic acids, L-quebrachitol, fatty acids have been reported to be the essential primary metabolites in the berries for plant growth. Earlier researches also revealed that the concentrations of reducing sugars (*e*.*g*., glucose and fructose) and organic compounds in plant are related to temperature, low temperature will induce reducing sugars and organic compounds accumulation^39, 40^. However, this result was not completely repeated in the present study, such as malic acid level were higher in *H*. *rhamnoides* ssp. *wolongensis* and *H*. *rhamnoides* ssp. *yunnanensis*, which distributed in the relative lowest temperature areas compared with other *Hippophaë* species/subspecies. *β*-D-glucose and *α*-D-glucose levels were higher in *H*. *rhamnoides* ssp. *mongolica*, which are mainly grown in relative higher temperature region.

The PCA and PLS-DA score plots showed that *H*. *rhamnoides* ssp. *sinensis* samples were projected in a larger region compared with other subspecies, indicating a larger intra-specific variation than other subspecies. This may due to the fact that the samples of *H*. *rhamnoides* ssp. *sinensis* were collected from a broader area, such as Sichuan, Qinghai, Tibet, and Gansu in China. These areas are parts of Qinghai-Tibet Plateau, which cover a vast geographic area with complicated landforms and various climate types^41, 42^. The different ecological factors, such as altitude, mean temperature of the warmest month, mean temperature of the coldest month, annual mean temperature, relative humidity, and precipitation may all be important to influence the primary metabolite concentrations^36, 43–46^. Therefore, these differences will reflect in PCA and PLS-DA score plots. Although intra-specific variations were observed in the score plots, the largest source of variation highlighted by the PCA and PLS-DA was inter-specific variation.

Additionally, *H*. *neurocarpa* samples were separated into two parts; one part grouped independently and the other was grouped with samples of *H*. *litangensis*. After a thorough analysis, it was found that the independent group of *H*. *neurocarpa* samples belonging to *H*. *neurocarpa* ssp. *neurocarpa*, and the samples that grouped together with *H*. *litangensis* samples were *H*. *neurocarpa* ssp. *stellatopilosa*. This finding is consistent with our early research, the relationship between the two species are very close with few variations based on the ITS2 and *psbA-trnH* sequences. In addition, *H*. *litangensis* shares a same haplotype with *H*. *neurocarpa* ssp. *stellatopilosa* based on ITS2 sequence^22^. Previous studies have also reported that the resource of *H*. *litangensis* is scarce with small distribution range, and mixed with *H*. *neurocarpa* ssp. *stellatopilosa* ^47^. Thus, we hypothesize that this mixed growth pattern and the similar genetic characteristics of *H*. *neurocarpa* ssp. *stellatopilosa* and *H*. *litangensis* resulted in the inseparable group in PCA and PLS-DA score plots.

Recent investigation have demonstrated that the metabolites, including quinic acid, malic acid, L-quebrachitol, *β*-D-glucose, *α*-D-glucose, fatty acids, and flavonoids possess various physiological activities, such as antioxidant^9, 48^, regulate the insulin and blood glucose levels^11^, and anti-carcinogenic^12^, cardioprotective, antifatigue, and anti-tussive effects^9, 49^. The results in this case confirm that these metabolites have great significance in the quality evaluation of sea buckthorn due to their strong power of species differentiation. Therefore, the present study suggests that these metabolic markers can be taken into account as the quality assessment index for a more comprehensively quality evaluation of sea buckthorn berries form different botanical origins.

In our earlier work, a series of experimental conditions were tested and an optimum condition was determined. The results demonstrated that three commonly used *Hippophe* species were differentiated and the interspecific discriminators were identified by ^1^H NMR based metabolomics^20^. Besides, ^1^H NMR based metabolomics were also used to distinguish and compare the metabolic profiles of two *H*. *rhamnoides* subspecies from Finland and China, some of the important metabolites were quantified by qNMR method, and the effect of the major ecological factors were also studied^14^. On the basis of the aforementioned publications, the present study took all seven species and seven subspecies native to China as research objects, optimized the previous experimental conditions and analytical method. The results proved that all the investigated species were successfully discriminated at species or subspecies level, and a total of 36 metabolites were identified from the ^1^H NMR spectra. Moreover, the important discriminators of sea buckthorn berries in a wide range of *Hippophaë* species have been identified and quantified through ^1^H NMR based metabolomics combined with multivariate statistical analysis. Therefore, this proposed method provides a basis for the traceability of sea buckthorn berries from different *Hippophaë* origins, and also offers an effective way to evaluate the qualities of sea buckthorn berry products.

In conclusion, the ^1^H NMR based metabolomics combined with multivariate statistical analysis was a fast and reliable method for species differentiation and quality control. The specific analysis of individual metabolites in the berries is crucial for quality assessment of natural products made from different *Hippophaë* species and subspecies for their specific therapeutic and health care effects. The PCA and PLS-DA models established in this study can be used as a *Hippophaë* mini-database for the unknown *Hippophaë* species recognition and authentication. We also believe that the broader application of ^1^H NMR based metabolomics in other edible herbs and herbal medicines may promote a safer market and greater consumer confidence by prevent plant origin counterfeiting.

## Methods

### Solvents and ch**e**micals

Methanol-*d*_4_ (CD_3_OD, 99.8%), deuterium oxide (D_2_O, 99.9%), chloroform-*d* (CDCl_3_, 99.8%), and dimethyl sulfoxide-*d*_6_ (DMSO-*d*_6_, 99.9%) were purchased from Cambridge Isotope Laboratories (Miami, FL, USA). 3-(Trimethylsilyl) propionic-2,2,3,3-*d*_4_ acid sodium salt (TSMP, 99%) was purchased from Sigma-Aldrich (St. Louis, Mo, USA). Monopotassium phosphate (KH_2_PO_4_, 99.5%) was obtained from Sinopharm Chemical Reagent Co., Ltd (Shanghai, China). The standard compounds were purchased from Ruifensi Biological Technology Co., Ltd. (Chengdu, China), Maisite Biological Technology Co., Ltd. (Chengdu, China) and Pusi Biological Technology Co., Ltd. (Chengdu, China). The detailed information were shown in Supplementary Table S2, and the structures of all standard compounds were unambiguously identified based on their ^1^H, ^13^C NMR spectral data, LC-MS data, and published literature^33–35, 50^.

### Sample collection and preparation

**Figure 5:**
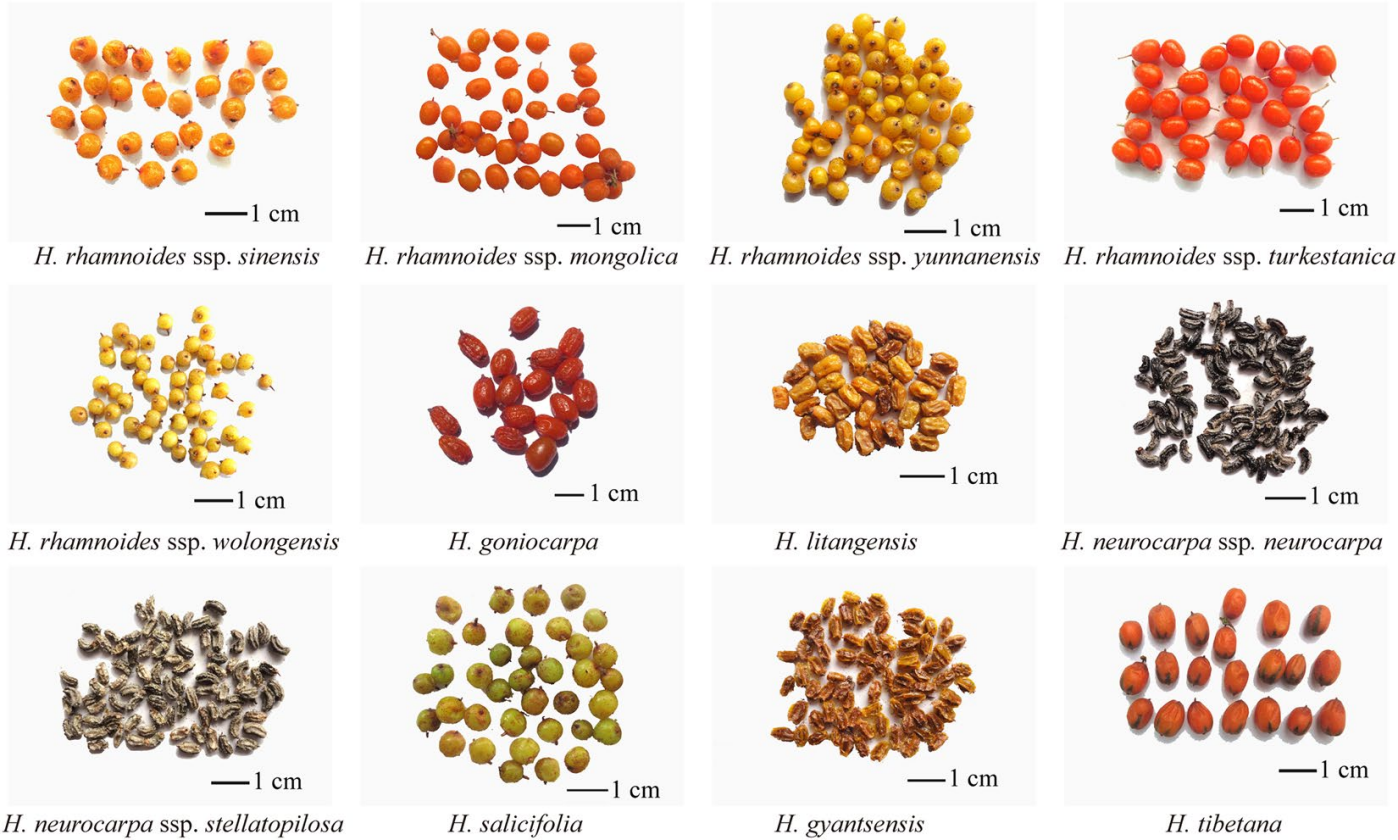
Berry samples from seven species and seven subspecies of *Hippophaë* collected from China.

A total of 90 sea buckthorn berry samples (Supplementary Table S3) representing seven species and seven subspecies (Fig. 5) were collected from the major producing areas in Sichuan, Qinghai, Xinjiang, Yunnan, and Tibet of China in July 2013. The samples were identified by Professor Yi Zhang, and voucher specimens were deposited in the College of Ethnic Medicine, Chengdu University of Traditional Chinese Medicine, China.

All the berries were oven-dried at 50 °C to completely eliminate moisture to constant weight. Then, samples were ground with liquid nitrogen using a SCIENTZ-48 (Scientz Bioscience Co., Inc., Zhejiang, China). Two hundred mg of each powdered sample were vortexed in 1.0 mL of CD_3_OD, and 0.3 mL pH 6.0 buffer comprising KH_2_PO_4_ in D_2_O (containing 0.04% TSMP as the internal chemical shift standard), which was then extracted by ultra-sonication for 30 min at room temperature. After extraction, the sample was centrifuged at 16,000 g for 5 min and subsequently filtered through a 0.45 μm membrane filter. Exactly 0.6 mL of filtrate was transferred into a standard 5 mm NMR tube for ^1^H NMR analysis.

### ^1^H NMR analysis and data processing

^1^H NMR spectra were recorded on a Bruker Avance 500 spectrometer (Bruker BioSpin, Rheinstetten, Germany), operating at a frequency of 500.15 MHz ^1^H and a temperature of 300 K, using a cryogenic triple-resonance probe and a Bruker automatic injector. A standard Bruker pulse sequence with water suppression (zgpr) was used to acquire ^1^H NMR spectra of sea buckthorn berry extracts, which were used for multivariate statistical analysis. For each sample, 128 transients were collected into 32 K data points using a spectral width of 7,500 Hz, an acquisition time of 4.369 s, a relaxation delay of 20 s, and the pulse width of 11.24 μs. A 0.3 Hz line-broadening function was applied to all spectra prior to Fourier transformation.

### Data processing

Fourier transformation, phase and baseline correction were applied to the data. Calibration of the data was carried out by shifting the TMSP signal to 0.0 ppm using TOPSPIN 2.1 software (Bruker Biospin GmbH, Rheinstetten, Germany). The NMR spectra were reduced to integrated buckets of equal width of 0.04 ppm each in the range of δ 10.5~0.5, excluding the residual signal of methanol (δ 3.26~3.34) and water (δ 4.74~5.02) using MestReNova software (version 6.1.0, Mestrelabs Research SL, Santiago de Compostela, Spain). All the integrated values were normalized to the intensity of the TMSP signal. The resulting data sets were then imported into SIMCA-P version 11.5 (Umetrics, Umeå, Sweden), and a mean-centered pre-process was applied for multivariate statistical analysis.

### Metabolite identification and quantification

The NMR signals were assigned and identified with the aid of published literature^14, 33–35, 51^ and the Spectral Database for Organic Compounds (http://sdbs.db.aist.go.jp). Additionally, the identifications were verified by carefully comparing the ^1^H NMR spectra of the standard compounds, and adding relevant standard compounds directly to pH-adjusted NMR samples^14, 27^.

The signal intensity in the ^1^H NMR spectrum is absolutely proportional to the molar concentration of metabolites^27, 28, 52^. Thus, the selected metabolites can be quantified through integration using TSMP as an internal standard according to equation (1):

1 $${m}_{X}={m}_{ST}\times (\frac{{A}_{X}}{{A}_{ST}})\times (\frac{M{W}_{X}}{M{W}_{ST}})\times (\frac{{N}_{ST}}{{N}_{X}})\ast $$

*: *m*_*X*_ is the unknown mass of the targeted metabolite; *m*_*ST*_ is the mass of the TMSP; *A*_*X*_ and *A*_*ST*_ are the integral areas for the selected signals; *MW*_X_ and *MW*_*ST*_ are the molecular weights of the targeted metabolite and TMSP; *N*_*X*_ and *N*_*ST*_ are the number of protons generating the integral signals.

### Statistical analysis

Principal component analysis (PCA) was used to give an intrinsic overview of the dataset and reveal possible groups and outliers^53^. A partial least squares discriminant analysis (PLS-DA) was also performed to maximize separation between groups^54^. Here, PCA and PLS-DA were conducted with mean-centered pre-process through SIMCA-P software (version 11.5, Umetrics, Umeå, Sweden). The metabolite concentration data were implemented by one-way analysis of variance (ANOVA, Version 18.0, SPSS Inc., Chicago, IL, USA). Differences between groups were assessed with Tukey’s multiple comparison tests, and the statistical significance was determined at *p* < 0.05^29^.

## Electronic supplementary material

Supplementary materials
