# Supplementary material for: Metabolic discrimination of sea buckthorn from different Hippophaë species by 1H NMR based metabolomics
Source: Sci Rep. 2017 May 8;7:1585. doi: 10.1038/s41598-017-01722-3 (PMC5431470; doi:10.1038/s41598-017-01722-3)
Supplement: Supplementary file 1 — Supplementary materials [file 41598_2017_1722_MOESM1_ESM.pdf]

Metabolic discrimination of sea buckthorn from different *Hippophaë* species by ^{1}H NMR based metabolomics

Yue Liu[1, 3†], Gang Fan[1†], Jing Zhang[1], Yi Zhang[1], Jingjian Li[4], Chao Xiong[5], Qi Zhang[2], Xiaodong Li[2*], Xianrong Lai[1*]

[1]College of Ethnic Medicine, Chengdu University of Traditional Chinese Medicine, Chengdu 611137, China

[2]National Institute for Food and Drug Control, Beijing 100050, China

[3]Sichuan Industrial Institute of Antibiotics, Chengdu University, Chengdu 610051, China

[4]College of Forestry and Landscape Architecture, South China Agricultural University, Guangzhou 510642, China

[5]College of Pharmacy, Hubei University of Chinese Medicine, Wuhan 430065, China

[†]These authors have contributed equally to this work.

[*]Correspondence: Xiaodong Li & Xianrong Lai

National Institute for Food and Drug Control

No. 2 Tiantan Xili

Beijing 100050, China

College of Ethnic Medicine, Chengdu University of Traditional Chinese Medicine

No. 1166 Liutai Ave

Chengdu 611137, China

xdli@nicpbp.org.cn (Xiaodong Li) & laixianrong@163.net (Xianrong Lai)

Supplementary figure legends

Figure S1 Representative ^{1}H NMR spectra of expanded regions and signal assignments. (1) sterols, (2) oleanolic acid, (3) saturated fatty acids, (4) unsaturated fatty acids, (5) D-fructose (6) isoleucine, (7) leucine, (8) valine, (9) alanie, (10) quinic acid, (11) malic acid, (12) L-queb rachitol, (13) dehydroascorbic acid, (14) sucrose, (15) uridine, (16) tryptophan, (17) histidine, (18) trigonelline, (19) *β*-D-glucose, (20) *α*-D-glucose, (21) asparagine, (22) quercetin, (23) kaem pferol, (24) isorhamnetin, (25) quercetin-3-*O*-*β*-D-rutinoside, (26) quercetin-3-*O*-*β*-D-glucoside, (2 7) isorhamnetin-3-*O*-*β*-D-rutinoside, (28) isorhamnetin-3-*O*-*β*-D-glucoside, (29) isorhamnetin-3-*O*-*β*-D-glucoside-7-*O*-*α*-L-rhamnoside, (30) quercetin-3-*O*-*β*-D-glucoside-7-*O*-*α*-L-rhamnoside, (31) is orhamnetin-3-*O*-*β*-D-galactoside-7-*O*-*α*-L-rhamnoside, (32) isorhamnetin-3-*O*-*α*-L-arabinopyranoside -7-*O*-*α*-L-rhamnoside, (33) kaempferol-3-*O*-*β*-D-sophoroside-7-*O*-*α*-L-rhamnoside, (34) isorhamneti n-3-*O*-*β*-D-sophoroside-7-*O*-*α*-L-rhamnoside, (35) quercetin-7-*O*-*α*-L-rhamnoside, and (36) isorha mnetin-7-*O*-*α*-L-rhamnoside.

Figure S2 PCA score plot of seven species and seven subspecies of *Hippophaë*.

Figure S3 The intercept values of the permutation plot of (A) five *H. rhamnoides* subspecies and (B) the remaining six *Hippophaë* species.

Figure S4 Multivariate statistical analysis of seven species and seven subspecies of *Hippop haë* only including aromatic region (6.5~9.5 ppm). (A) PCA score plot of five *H. rhamnoides* subspecies, (B) PCA score plot of the remaining six *Hippophaë* species, (C) PLS-DA score pl ot of five *H. rhamnoides* subspecies, (D) PLS-DA score plot of the remaining six *Hippophaë* spe cies. (E) PLS-DA loading plots of five *H. rhamnoides* subspecies, (F) PLS-DA loading plots of the remaining six *Hippophaë* species, (G) the intercept values of the permutation plot of five *H. rhamnoides* subspecies, (H) the intercept values of the permutation plot of the remaining six *Hippophaë* species. Numbers in Fig. S4 E and F are (1) quercetin, (2) kaempferol, (3) isorh amnetin, (4) quercetin-3-*O*-*β*-D-rutinoside, (5) quercetin-3-*O*-*β*-D-glucoside, (6) isorhamnetin-3-*O*-*β*-D-rutinoside, (7) isorhamnetin-3-*O*-*β*-D-glucoside, (8) isorhamnetin-3-*O*-*β*-D-glucoside-7-*O*-*α*-L-r hamnoside, (9) quercetin-3-*O*-*β*-D-glucoside-7-*O*-*α*-L-rhamnoside, (10) isorhamnetin-3-*O*-*β*-D-galac toside-7-*O*-*α*-L-rhamnoside, (11) isorhamnetin-3-*O*-*α*-L-arabinopyranoside-7-*O*-*α*-L-rhamnoside, (12) kaempferol-3-*O*-*β*-D-sophoroside-7-*O*-*α*-L-rhamnoside, (13) isorhamnetin-3-*O*-*β*-D-sophoroside-7-*O*-*α*-L-rhamnoside, and (14) quercetin-7-*O*-*α*-L-rhamnoside.

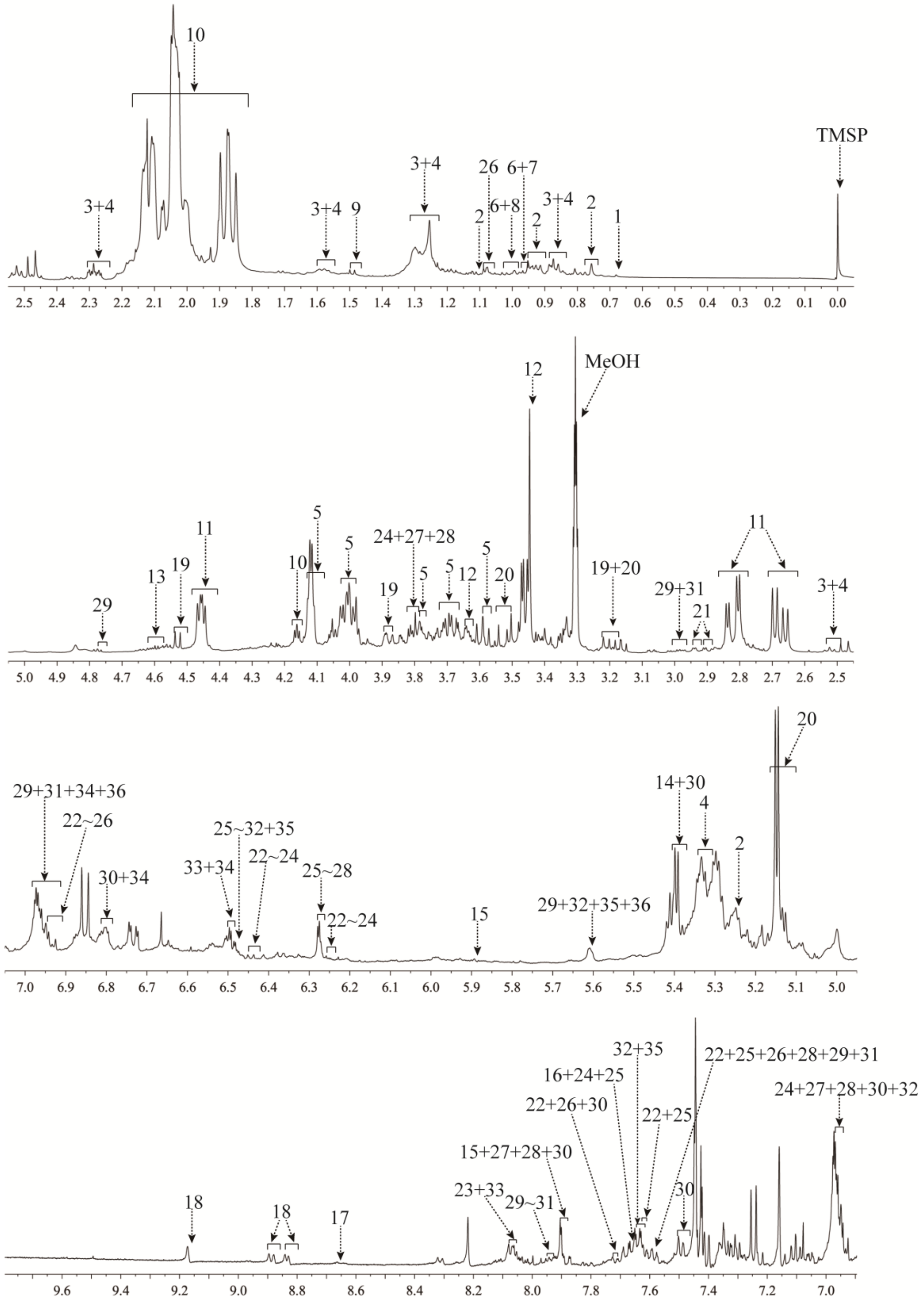

Figure S1 Representative ^{1}H NMR spectra of expanded regions and signal assignments. (1) sterols, (2) oleanolic acid, (3) saturated fatty acids, (4) unsaturated fatty acids, (5) D-fructose (6) isoleucine, (7) leucine, (8) valine, (9) alanie, (10) quinic acid, (11) malic acid, (12) L-queb rachitol, (13) dehydroascorbic acid, (14) sucrose, (15) uridine, (16) tryptophan, (17) histidine,

(18) trigonelline, (19) β-D-glucose, (20) α-D-glucose, (21) asparagine, (22) quercetin, (23) kaempferol, (24) isorhamnetin, (25) quercetin-3-*O*-β-D-rutinoside, (26) quercetin-3-*O*-β-D-glucoside, (27) isorhamnetin-3-*O*-β-D-rutinoside, (28) isorhamnetin-3-*O*-β-D-glucoside, (29) isorhamnetin-3-*O*-β-D-glucoside-7-*O*-α-L-rhamnoside, (30) quercetin-3-*O*-β-D-glucoside-7-*O*-α-L-rhamnoside, (31) isorhamnetin-3-*O*-β-D-galactoside-7-*O*-α-L-rhamnoside, (32) isorhamnetin-3-*O*-α-L-arabinopyranoside-7-*O*-α-L-rhamnoside, (33) kaempferol-3-*O*-β-D-sophoroside-7-*O*-α-L-rhamnoside, (34) isorhamnetin-3-*O*-β-D-sophoroside-7-*O*-α-L-rhamnoside, (35) quercetin-7-*O*-α-L-rhamnoside, and (36) isorhamnetin-7-*O*-α-L-rhamnoside.

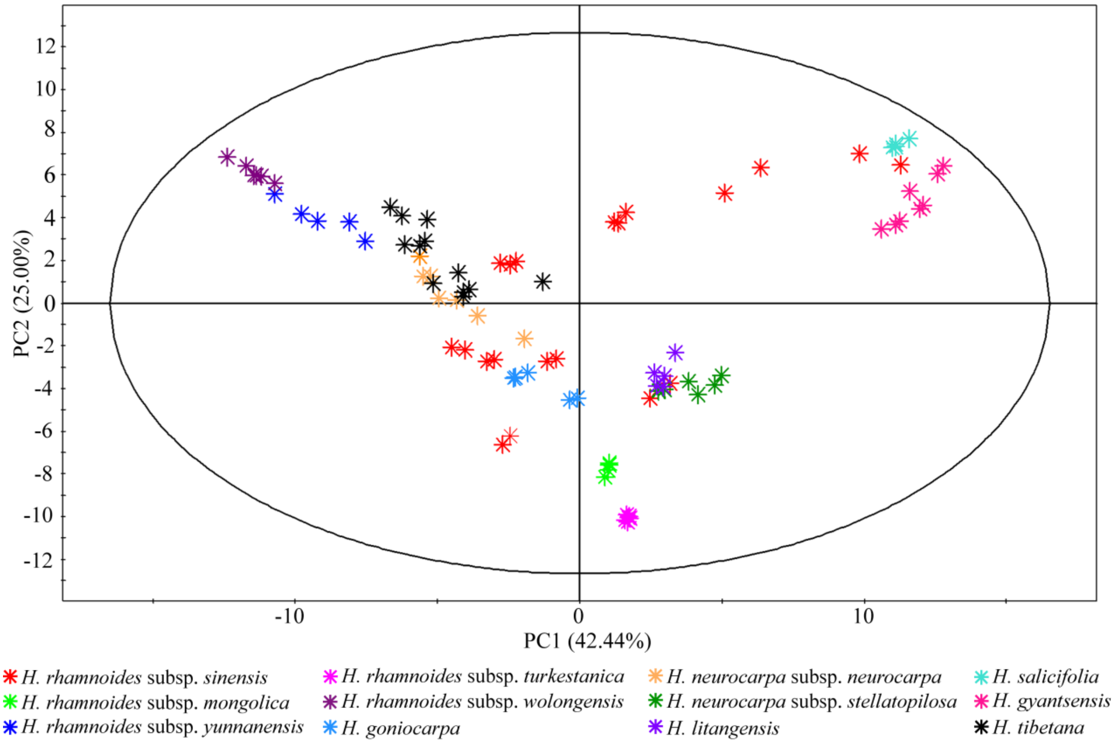

Figure S2 PCA score plot of seven species and seven subspecies of *Hippophaë*.

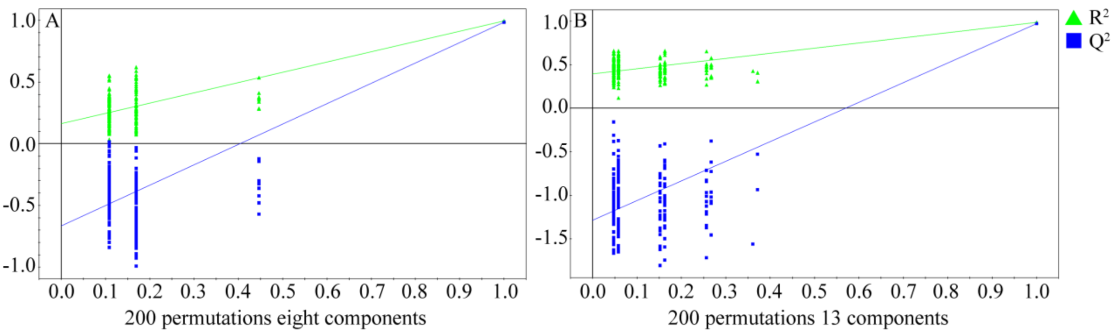

Figure S3 The intercept values of the permutation plot of (A) five *H. rhamnoides* subspecies and

(B) the remaining six *Hippophaë* species.

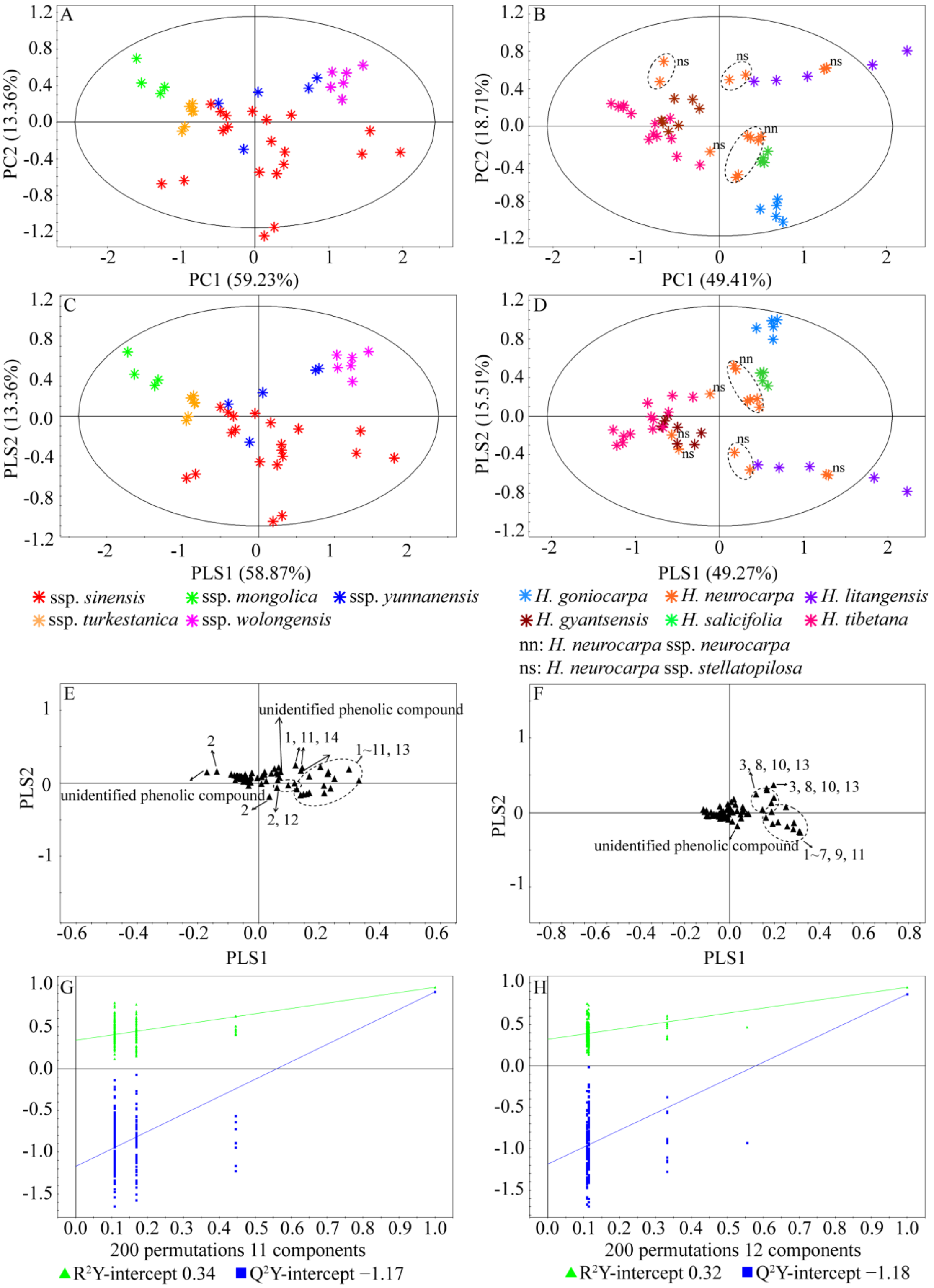

Figure S4 Multivariate statistical analysis of seven species and seven subspecies of *Hippophaë* only including aromatic region (6.5~9.5 ppm). (A) PCA score plot of five *H. rhamnoides* subspecies, (B) PCA score plot of the remaining six *Hippophaë* species, (C) PLS-DA score pl

ot of five *H. rhamnoides* subspecies, (D) PLS-DA score plot of the remaining six *Hippophaë* species. (E) PLS-DA loading plots of five *H. rhamnoides* subspecies, (F) PLS-DA loading plots of the remaining six *Hippophaë* species, (G) the intercept values of the permutation plot of five *H. rhamnoides* subspecies, (H) the intercept values of the permutation plot of the remaining six *Hippophaë* species. Numbers in Fig. S4 E and 4F are (1) quercetin, (2) kaempferol, (3) isorhamnetin, (4) quercetin-3-*O*-*β*-D-rutinoside, (5) quercetin-3-*O*-*β*-D-glucoside, (6) isorhamnetin-3-*O*-*β*-D-rutinoside, (7) isorhamnetin-3-*O*-*β*-D-glucoside, (8) isorhamnetin-3-*O*-*β*-D-glucoside-7-*O*-*α*-L-rhamnoside, (9) quercetin-3-*O*-*β*-D-glucoside-7-*O*-*α*-L-rhamnoside, (10) isorhamnetin-3-*O*-*β*-D-galactoside-7-*O*-*α*-L-rhamnoside, (11) isorhamnetin-3-*O*-*α*-L-arabinopyranoside-7-*O*-*α*-L-rhamnoside, (12) kaempferol-3-*O*-*β*-D-sophoroside-7-*O*-*α*-L-rhamnoside, (13) isorhamnetin-3-*O*-*β*-D-sophoroside-7-*O*-*α*-L-rhamnoside, and (14) quercetin-7-*O*-*α*-L-rhamnoside.

Supplementary tables

Table S1 Concentration of eight selected metabolites in the berries of different *Hippophaë* species (mg/ g fresh weight, mean ± std). Different superscript letters indicate significant differences between species, and the same superscript letters indicate no significant differences between species based on Tukey's multiple comparison tests ($p < 0.05$).

Species	Oleanolic acid	Alanine	Quinic acid	Fatty acids	Malic acid	L-quebrachitol	β-D-glucose	α-D-glucose
H. rhamnoides ssp. *sinensis* (*n* = 20)	0.127 ± 0.056^{b}	0.114 ± 0.046^{bcdf}	10.306 ± 9.447^{b}	10.326 ± 11.881^{bc}	21.749 ± 9.982^{bc}	5.123 ± 2.071^{bdef}	5.849 ± 6.892^{b}	3.229 ± 5.259^{b}
H. rhamnoides ssp. *mongolica* (*n* = 4)	0.057 ± 0.005^{b}	0.059 ± 0.025^{bdf}	9.871 ± 0.343^{bc}	1.626 ± 0.146^{bc}	5.497 ± 0.273^{b}	7.639 ± 0.343^{ad}	34.818 ± 1.436^{a}	27.100 ± 2.199^{a}
H. rhamnoides ssp. *yunnanensis* (*n* = 5)	0.190 ± 0.067^{ab}	0.167 ± 0.032^{ac}	5.039 ± 2.142^{bc}	3.627 ± 1.104^{bc}	53.991 ± 6.237^{ac}	4.251 ± 0.569^{bdehi}	4.205 ± 1.1458^{b}	0.526 ± 0.139^{b}
H. rhamnoides ssp. *turkestanica* (*n* = 6)	0.089 ± 0.020^{b}	0.046 ± 0.046^{bf}	35.869 ± 9.013^{a}	3.308 ± 1.351^{bc}	14.594 ± 6.944^{bd}	7.739 ± 3.143^{a}	3.663 ± 2.184^{b}	2.061 ± 1.523^{b}
H. rhamnoides ssp. *wolongensis* (*n* = 6)	0.116 ± 0.033^{bc}	0.127 ± 0.021^{af}	0.825 ± 0.245^{bc}	1.678 ± 0.363^{b}	57.964 ± 4.584^{a}	3.237 ± 0.287^{bglm}	4.517 ± 1.272^{b}	0.705 ± 0.103^{b}
H. goniocarpa (*n* = 5)	0.104 ± 0.032^{bc}	0.128 ± 0.023^{af}	14.151 ± 1.477^{b}	4.460 ± 0.843^{bc}	20.159 ± 6.178^{bd}	6.636 ± 1.014^{ae}	2.072 ± 0.150^{b}	0.565 ± 0.250^{b}
H. litangensis (*n* = 8)	0.153 ± 0.017^{ab}	0.187 ± 0.028^{ac}	0.572 ± 0.147^{bc}	2.353 ± 1.088^{bc}	9.065 ± 2.073^{b}	3.396 ± 0.428^{bejk}	1.217 ± 0.248^{b}	0.697 ± 0.256^{b}
H. neurocarpa ssp. *neurocarpa* (*n* = 5)	0.185 ± 0.045^{ab}	0.197 ± 0.065^{a}	2.079 ± 2.768^{bc}	3.453 ± 1.761^{bc}	20.328 ± 8.786^{bd}	4.787 ± 0.867^{aeg}	3.602 ± 3.791^{b}	0.386 ± 0.167^{b}
H. neurocarpa ssp. *stellatopilosa* (*n* = 6)	0.213 ± 0.021^{ac}	0.175 ± 0.013^{ac}	2.487 ± 2.305^{bc}	3.564 ± 1.234^{b}	22.503 ±15.874^{bd}	3.396 ± 0.647^{bejk}	1.432 ± 0.529^{b}	0.337 ± 0.181^{b}
H. salicifolia (*n* = 4)	0.089 ± 0.016^{bc}	0.122 ± 0.022^{af}	0.743 ± 0.158^{bc}	30.137 ± 5.747^{a}	4.912 ± 0.819^{b}	1.008 ± 0.167^{chjl}	0.524 ± 0.119^{b}	0.292 ± 0.056^{b}
H. gyantsensis (*n* = 8)	0.235 ± 0.041^{a}	0.152 ± 0.059^{ad}	0.547 ± 0.304^{bc}	18.429 ± 9.976^{abc}	9.740 ± 6.270^{b}	4.934 ± 0.976^{afikm}	0.833 ± 0.495^{b}	0.431 ± 0.238^{b}
H. tibetana (*n* = 13)	0.253±0.119^{a}	0.143 ± 0.063^{ad}	0.292 ± 0.133^{c}	19.768 ± 11. 527^{ac}	35.733 ± 7.066^{acd}	7.091 ± 1.970^{a}	6.825 ± 5.972^{b}	4.425 ± 4.220^{b}

Table S2 Standard compounds used in this study.

No.	Chemical reference substance	Voucher No.	Source
1	Quercetin	H-009-130126	Ruifensi Biological Technology Co., Ltd. (Chengdu, China)
2	Kaempferol	S-064-130518	Ruifensi Biological Technology Co., Ltd. (Chengdu, China)
3	Isorhamnetin	Y-039-130326	Ruifensi Biological Technology Co., Ltd. (Chengdu, China)
4	Isorhamnetin-3-*O*-*β*-D-rutinoside	S-063-130114	Ruifensi Biological Technology Co., Ltd. (Chengdu, China)
5	Quercetin-3-*O*-*β*-D-rutinoside	MUST-13040302	Maisite Biological Technology Co., Ltd. (Chengdu, China)
6	Quercetin-3-*O*-*β*-D-glucoside	MUST-13021811	Maisite Biological Technology Co., Ltd. (Chengdu, China)
7	Isorhamnetin-3-*O*-*β*-D-glucoside	MUST-13022011	Maisite Biological Technology Co., Ltd. (Chengdu, China)
8	Oleanolic acid	MUST-13041606	Maisite Biological Technology Co., Ltd. (Chengdu, China)
9	Kaempferol-3-*O*-*β*-D-sophoroside-7-*O*-*α*-L- rhamnoside	PS140926-01	Pusi Biological Technology Co., Ltd. (Chengdu, China)
10	Isorhamnetin-3-*O*-*β*-D-sophoroside-7-*O*-*α*-L-rhamnoside	PS140926-02	Pusi Biological Technology Co., Ltd. (Chengdu, China)
11	Isorhamnetin-3-*O*-*β*-D-glucoside-7-*O*-*α*-L-rhamnoside	PS140926-03	Pusi Biological Technology Co., Ltd. (Chengdu, China)
12	Quercetin-3-*O*-*β*-D-glucoside-7-*O*-*α*-L-rhamnoside	PS140926-04	Pusi Biological Technology Co., Ltd. (Chengdu, China)
13	Isorhamnetin-3-*O*-*β*-D-galactoside-7-*O*-*α*-L-rhamnoside	PS140927-01	Pusi Biological Technology Co., Ltd. (Chengdu, China)
14	Quercetin-7-*O*-*α*-L-rhamnoside	PS140927-02	Pusi Biological Technology Co., Ltd. (Chengdu, China)
15	Isorhamnetin-7-*O*-*α*-L-rhamnoside	PS140927-03	Pusi Biological Technology Co., Ltd. (Chengdu, China)
16	Isorhamnetin-3-*O*-*α*-D-arabinopyranoside-7-*O*-*α*-L-rhamnoside	PS140927-04	Pusi Biological Technology Co., Ltd. (Chengdu, China)

Table S3 Plant materials used in this study.

Latin Name	Voucher No.	Location
H. rhamnoides ssp. *sinensis*	YC0546MT01	Wanlin, Jinchuan, Sichuan, China
	YC0546MT02	Maierma, Aba, Sichuan, China
	YC0546MT03	Shili, Songpan, Sichuan, China
	YC0546MT04	Rongrida, Rangtang, Sichuan, China
	YC0546MT05	Nanmenxia, Huzhu, Qinghai, China
	YC0546MT06	Puxi, Lixian, Sichuan, China
	YC0546MT07	Chaka, Wulan, Qinghai, China
	YC0546MT08	Gatuo, Mangkang, Tibet, China
	YC0546MT09	Aba, Aba, Sichuan, China
	YC0546MT10	Luoerda, Aba, Sichuan, China
	YC0546MT11	Kehe, Aba, Sichuan, China
	YC0546MT12	Nawu, Hezuo, Gansu, China
	YC0546MT13	Yala, Kangding, Sichuan, China
	YC0546MT14	Chuanzhusi, Songpan, Sichuan, China
	YC0546MT15	Rilong, Xiaojin, Sichuan, China
	YC0546MT16	Fubian, Xiaojin, Sichuan, China
	YC0546MT17	Dawei, Xiaojin, Sichuan, China
	YC0546MT18	Jiazhulin, Gongga, Sichuan, China
	YC0546MT19	Xianghua, Datong, Qinghai, China
	YC0546MT20	Cunge, Litang, Sichuan, China
H. rhamnoides ssp. *mongolica*	YC0547MT01~04	Buerjin, Altay, Xinjiang, China
H. rhamnoides ssp. *yunnanensis*	YC0548MT01	Guxiang, Bomi, Tibet, China
	YC0548MT02	Rewa, Milin, Tibet, China
	YC0548MT03	Shangri-la, Diqing, Yunnan, China
	YC0548MT04	Jiawa, Litang, Sichuan, China
	YC0548MT05	Chitu, Daocheng, Yunnan, China
H. rhamnoides ssp. *turkestanica*	YC0549MT01~03	Tulin, Zhada, Tibet, China
	YC0549MT04~06	Aotebeixi, Wushi, Xinjiang, China
H. rhamnoides ssp. *wolongensis*	YC0550MT01~03	Taiping, Maoxian, Sichuan, China
	YC0550MT04~06	Puxi, Lixian, Sichuan, China
H. goniocarpa	YC0551MT01~05	Galitai, Songpan, Sichuan, China
H. litangensis	YC0552MT01~05	Jiawa, Litang, Sichuan, China
H. neurocarpa ssp. *neurocarpa*	YC0553MT01	Babao, Qilian, Qinghai, China
	YC0553MT02	Jiawa, Litang, Sichuan, China
	YC0553MT03	Jiawa, Litang, Sichuan, China
	YC0553MT04	Chali, Aba, Sichuan, China
	YC0553MT05	Maierma, Aba, Sichuan, China
	YC0553MT06	Babao, Qilian, Qinghai, China
H. neurocarpa ssp. *stellatopilosa*	YC0554MT01~05	Gaocheng, Litang, Sichuan, China
	YC0554MT06~08	Benge, Litang, Sichuan, China

H. salicifolia	YC0653MT01~04	Lebu, Cuona, Tibet, China
H. gyantsensis	YC0654MT01	Qiangna, Milin, Tibet, China
	YC0654MT02	Jieba, Naidong, Tibet, China
	YC0654MT03	Ridang, Longzi, Tibet, China
	YC0654MT04	Gangtui, Gongga, Tibet, China
	YC0654MT05	Pozhang, Naidong, Tibet, China
	YC0654MT06	Jiaxing, Gongbujiangda, Tibet, China
	YC0654MT07	Mozhugongka, Mozhugongka, Tibet, China
	YC0654MT08	Jiubu, Linzhi, Tibet, China
H. tibetana	YC0655MT01	Langkazi, Langkazi, Tibet, China
	YC0655MT02	Duoma, Ruoergai, Sichuan, China
	YC0655MT03	Tangke, Ruoergai, Sichuan, China
	YC0655MT04	Riduo, Mozhugongka, Tibet, China
	YC0655MT05	Jiangrong, Hongyuan, Sichuan, China
	YC0655MT06	Maiwa, Hongyuan, Sichuan, China
	YC0655MT07	Nanmenxia, Huzhu, Qinghai, China
	YC0655MT08	Tawa, Ruoergai, Sichuan, China
	YC0655MT09	Chali, Aba, Sichuan, China
	YC0655MT10	Longriba, Hongyuan, Sichuan, China
	YC0655MT11	Keluodong, Dege, Sichuan, China
	YC0655MT12	Waqie, Hongyuan, Sichuan, China
	YC0655MT13	Anqu, Hongyuan, Sichuan, China
